# The Anti-Influenza Virus Drug Favipiravir Has Little Effect on Replication of SARS-CoV-2 in Cultured Cells

**DOI:** 10.1128/AAC.00020-21

**Published:** 2021-04-19

**Authors:** Yuriko Tomita, Makoto Takeda, Shutoku Matsuyama

**Affiliations:** a Department of Virology III, National Institute of Infectious Diseases, Tokyo, Japan

**Keywords:** COVID-19, SARS-CoV-2, T-705, Avigan, drug, favipiravir, influenza

## LETTER

Favipiravir (T-705, commercial name Avigan), a drug developed to treat influenza virus infection, has been used in some countries as an oral treatment for COVID-19; however, its clinical efficacy in this context is controversial. The anti-SARS-CoV-2 effects of favipiravir reported by previous studies are inconsistent. For example, the findings of Jeon et al. reported in this journal (1) and others (2) demonstrate that favipiravir (500 μM) shows negligible effects against SARS-CoV-2 in cultured cells, whereas two other studies reported weak effects, with a 50% effective concentration (EC_50_) ranging from 61.88 to 207.1 μM (3, 4). These discrepancies may result from differences in the assay protocol used.

**FIG 1:**
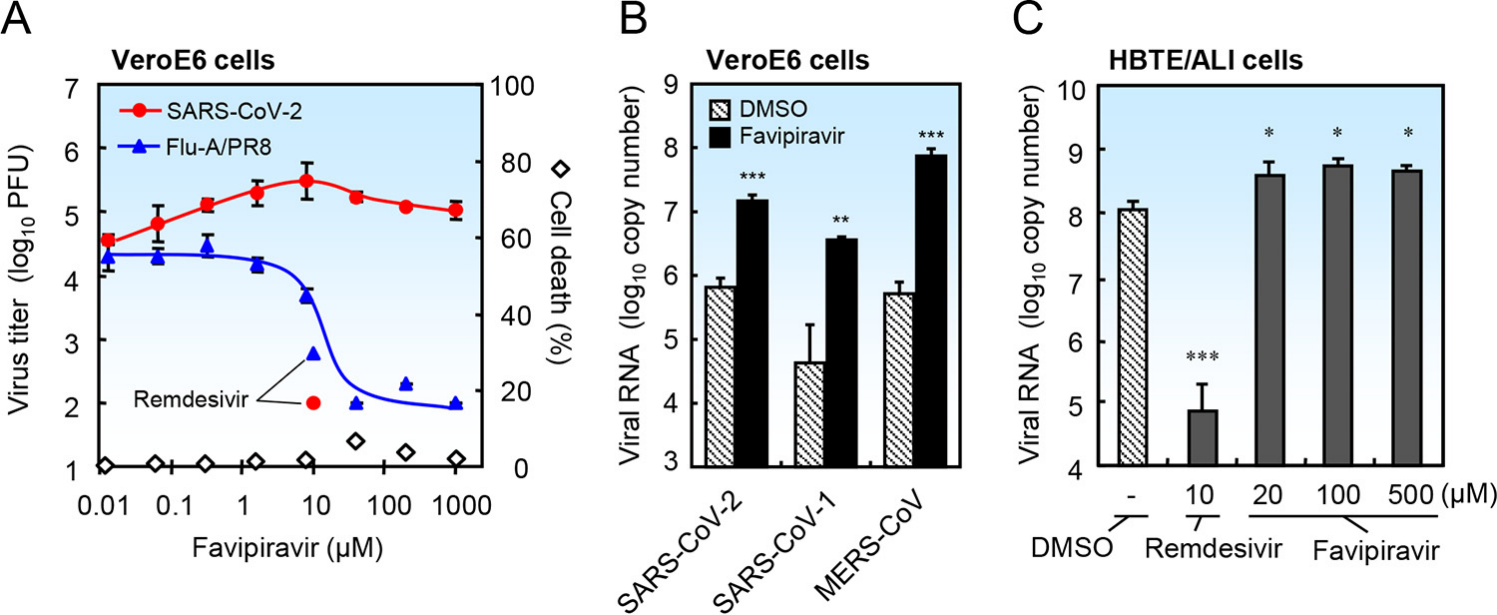
Favipiravir does not block replication of SARS-CoV-2 in cultured cells. To minimize the effects of the drug solvent, 400 mM favipiravir (23384, Cayman Chemical) was prepared in dimethyl sulfoxide (DMSO) as a stock solution and diluted >400-fold in medium before use. (A) VeroE6 cells seeded in 96-well plates were infected with SARS-CoV-2 (strain WK-521) or influenza A virus (strain PR8) at an MOI (multiplicity of infection) of 0.1 in the presence of DMSO or favipiravir. To prime influenza virus and SARS-CoV-2 for infection, 1 μg/ml trypsin was added to the medium. After incubation for 2 days, the culture media were collected and the virus titer of SARS-CoV-2 or influenza virus was measured by a plaque assay using VeroE6/TMPRSS2 cells (5) or MDCK cells, respectively. Data represent the average of three independent experiments (*n* = 3). Average cell death in the absence of virus was measured in a WST assay (*n* = 4). (B) VeroE6 cells were infected with SARS-CoV-2 (strain WK-521), SARS-CoV-1 (strain Frankfurt), or MERS-CoV (strain EMC) at an MOI of 0.1 in the presence of DMSO or favipiravir (8 μM), and then incubated for 2 days. Trypsin was not added to the culture medium. Viral RNA was extracted from the culture medium and quantified by real-time PCR using the SARS-2-E, SARS-N, and MERS-upE primer/probe sets (*n* = 4) (11, 12). (C) Differentiated human bronchial tracheal epithelial cells (HBTE/ALI cells) were infected with SARS-CoV-2 at an MOI of 0.01 in the presence of DMSO or favipiravir, and then incubated for 3 days. Viral RNA was extracted from cells and quantified by real-time PCR using the SARS-2-E primer/probe set (11). Data are presented as the mean ± standard deviation (SD) (*n* = 4). Two-tailed Student’s *t* tests were used to analyze statistical significance compared with the DMSO control: *, significant (*P ≤ *0.05); **, highly significant (*P ≤ *0.01); and ***, very highly significant (*P ≤ *0.001).

Here, we compared the effects of favipiravir on replication of SARS-CoV-2 and influenza virus in VeroE6 cells by quantifying the amount of propagated virus in medium via a plaque assay (5). Favipiravir blocked propagation of influenza virus in a concentration-dependent manner; however, it actually enhanced that of SARS-CoV-2 (Fig. 1A). Favipiravir significantly enhanced viral RNA replication in culture medium of VeroE6 cells infected with SARS-CoV-2, SARS-CoV-1, or MERS-CoV (Fig. 1B). Furthermore, favipiravir at 20 to 500 μM slightly, but significantly, enhanced RNA replication of SARS-CoV-2 in differentiated primary human bronchial tracheal epithelial cells cultured at an air-liquid interface (HBTE/ALI cells) (Fig. 1C). Favipiravir can be converted into favipiravir-ribofuranosyl-5′-triphosphate in cells and may influence cellular nucleoside/nucleotide metabolism, which may affect viral replication.

A recent study using hamsters revealed that the effective dose of favipiravir required to suppress replication of SARS-CoV-2 is 1.0 g/kg body weight, administered by intraperitoneal (i.p.) injection (6). Data from another group suggest that hamsters lost 20% of their body weight after i.p. injection of favipiravir at a dose of about 1.0 g/kg body weight (7). Such a high dose may not be practical for use in humans; however, high plasma trough concentrations of favipiravir were reported in clinical trials in Ebola-infected patients. In that study, favipiravir was given orally at a dose of 6 g or 2.4 g/day, after which the median observed trough concentration in blood plasma was 46.1 μg/ml (293 μM) (8). Nevertheless, we found that this concentration was totally ineffective; rather, it was counterproductive, as mentioned above. Recently, the manufacturer reported the results of its own clinical trials showing that symptoms of COVID-19 in a favipiravir-treated group improved after 11.9 days compared with 14.7 days in a placebo-treated group (9). So far, we are unable to provide a scientific rationale for the improved clinical symptoms after treatment with favipiravir.

Regardless of the data presented above, we feel compelled to raise awareness about administration of favipiravir to pregnant women; this is contraindicated due to the known teratogenic side effects of the drug (10).

The pressures brought to bear on societies by the COVID-19 pandemic mean that we may make poor judgments in the hope of identifying a “wonder” drug. Thus, we implore that drug approval is always handled in a manner based on scientific evidence.
